# Re-Addressing Dementia by Network Medicine and Mechanism-Based Molecular Endotypes

**DOI:** 10.3233/JAD-230694

**Published:** 2023-10-24

**Authors:** Mayra Pacheco Pachado, Ana I. Casas, Mahmoud H. Elbatreek, Cristian Nogales, Emre Guney, Alberto J. Espay, Harald H.H.W. Schmidt

**Affiliations:** a Department of Pharmacology and Personalised Medicine, School of Mental Health and Neuroscience, Maastricht University, Maastricht, The Netherlands; b Universitätsklinikum Essen, Klinik für Neurologie, Essen, Germany; c Department of Pharmacology and Toxicology, Faculty of Pharmacy, Zagazig University, Zagazig, Egypt; d Discovery and Data Science (DDS) Unit, STALICLA R&D SL, Barcelona, Spain; e James J. and Joan A. Gardner Family Center for Parkinson’s Disease and Movement Disorders, Department of Neurology, University of Cincinnati, Cincinnati, OH, USA

**Keywords:** Alzheimer’s disease, dementia, endophenotypes, precision medicine, protein-protein interaction network, systems medicine

## Abstract

Alzheimer’s disease (AD) and other forms of dementia are together a leading cause of disability and death in the aging global population, imposing a high personal, societal, and economic burden. They are also among the most prominent examples of failed drug developments. Indeed, after more than 40 AD trials of anti-amyloid interventions, reduction of amyloid-β (Aβ) has never translated into clinically relevant benefits, and in several cases yielded harm. The fundamental problem is the century-old, brain-centric phenotype-based definitions of diseases that ignore causal mechanisms and comorbidities. In this hypothesis article, we discuss how such current outdated nosology of dementia is a key roadblock to precision medicine and articulate how Network Medicine enables the substitution of clinicopathologic phenotypes with molecular endotypes and propose a new framework to achieve precision and curative medicine for patients with neurodegenerative disorders.

## INTRODUCTION

Drug discovery is in a conceptual crisis. The landscape of chronic diseases has a glaring scarcity of curative, causal mechanism-based approaches. A clear illustration of this can be seen in Alzheimer’s disease (AD) and other forms of dementia such as vascular dementia, dementia with Lewy bodies (DLB), and Parkinson’s disease dementia (PDD), which despite countless hypotheses on possible pathomechanisms remain untreatable. One possible source of failure is the stubborn clinging to the one disease-one target-one drug dogma.

Virtually all existing drug development programs for dementia have been based on the “proteinopathy” concept, whereby proteins in an aggregated state (a cross-beta sheet configuration also known as amyloid, such as amyloid-β (Aβ) plaques in AD and Lewy bodies in DLB and PDD) shall *cause* the disease. As a consequence of this toxic gain-of-function concept of aggregated proteins causing neurodegenerative diseases, removing them from the brain should cure these diseases. The recent accelerated approval of the anti-amyloid monoclonal antibodies aducanumab and lecanemab by the US Food and Drug Administration became the culmination of this paradigm. The cumulative lessons of more than 40 anti-Aβ Alzheimer’s trials should have taught us otherwise: 15 monoclonal anti-amyloid antibodies significantly reduced amyloid and, surprisingly, significantly worsened patients’ outcomes compared to placebo. None of these trials was interpreted as a rejection of the hypothesis. Instead, the 16th and 17th anti-amyloid antibodies, lecanemab [1] and donanemab [2], which met the statistical threshold in the opposite direction, have been taken as a confirmation of the hypothesis [3], although this never translated into clinically relevant benefits [4]. Instead, reduction in soluble Aβ levels, as measured in cerebrospinal fluid, is harmful to humans, and removal of insoluble Aβ may lead to microhemorrhages, brain atrophy, and death [5, 6]. The toxic Aβ hypothesis has become virtually unfalsifiable [7].

Aβ, however, has important physiological roles, including neuroprotection. Thus, the loss of the soluble and therefore functional form of Aβ, i.e., a *proteinopenia*, may equally explain cognitive deficits and atrophy and possibly better than any particular plaque load, even among carriers of autosomal dominant AD-causing amyloid-beta precursor protein (*APP*), presenilin 1 (*PSEN1*), and presenilin 2 (*PSEN2*) mutations [8]. Many alternative causal hypotheses for dementia have been proposed; however, in the face of a “resilient” amyloid hypothesis, these have not received adequate support or buy-in to be tested [9–13]. Collective evidence should have sufficed to consider amyloid rather a downstream consequence in cellular pathophysiology, a sign of a range of biological stressors, not their cause.

**Fig. 1:**
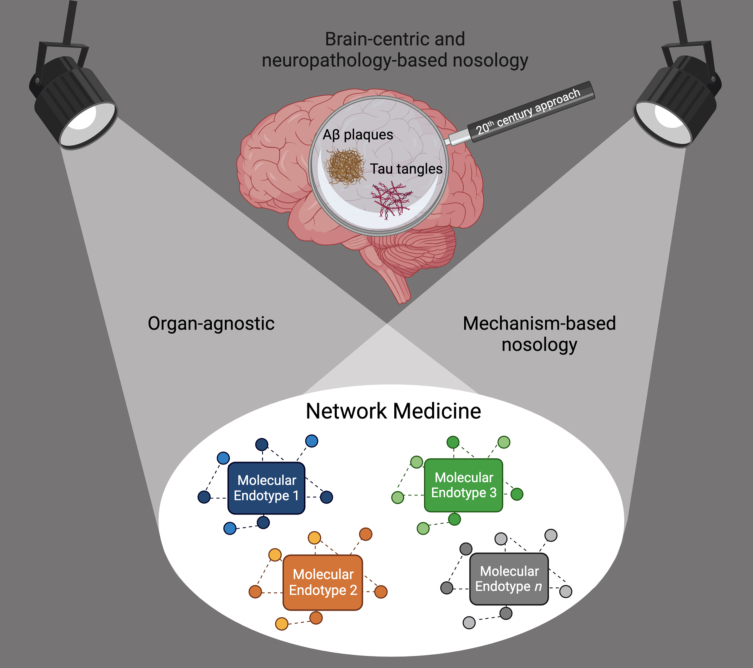
Shift from a brain-centric and neuropathology-based dementia nosology towards an organ-agnostic, mechanism-based nosology. Network Medicine accounts for causal heterogeneity and the identification of the molecular endotypes leading to the syndrome of dementia.

Thus, the field of dementia—as those of many other chronic diseases—is in need of a paradigm shift to distance itself from using symptoms and pathology in organs as anchoring disease definitions. We call for a move from the current convergent clinico-pathologic towards a divergent, organ-agnostic, and mechanism-based disease nosology (Fig. 1). Even rare monogenic diseases cause symptoms in more than one organ, thus organ-based taxonomies make little sense and likely obstruct innovation.

## THE CURRENT OUTDATED FRAMEWORK TO DIAGNOSE AND TREAT DEMENTIA

The diagnosis of AD, DLB, PDD, PD, and other neurodegenerative disorders have been based on the combination of clinical descriptions and post-mortem microscopic observations, the limited technologies available in the 19th century. Even before Alois Alzheimer described the famous case of Auguste Deter in 1906, the prevailing approach was, and still is, to artificially separate pathological conditions within the same patient to be either in or outside the brain and define them as distinct diseases. Prolific work was carried on by Alois Alzheimer and contemporary psychiatrists in their search to define the links between biology and a plethora of mental disorders. The focus, however, has always been within the brain. This brain-centric approach has been maintained by the majority of clinicians and researchers to date, but now halts conceptual progress in understanding, preventing, and treating dementia.

A single pathology in the brain is the exception rather than the rule. Real-world aging individuals, with or without dementia, have mixed manifestations of neuropathological markers in their brains, such as AD markers, Lewy bodies, transactive response DNA binding protein 43 kDa (TDP-43) inclusions, and vascular pathologies, with almost 80% of these individuals presenting with at least two of such neuropathologies [14]. And in contrast with the model of brain-centricity, these “dementia specific” markers can be often found in organs outside the brain [15], as for example Aβ aggregates documented in skin, intestines [16], heart [17], and pancreas [18], with hyperphosphorylated tau also found in the last two.

Moreover, in clinical practice very rarely we find patients with dementia without any other concurrent diseases. Of all comorbidities, vascular disease and metabolic abnormalities stand out and are frequently observed preceding dementia [19–21]. More often than not, AD patients show morphological substrates of cerebrovascular disease with matching risk factors such as hypertension and diabetes, pointing to the existence of shared pathobiological mechanisms with well-defined genetic and molecular underpinnings [22–28].

## A NEW FRAMEWORK FOR DEMENTIA BY NETWORK MEDICINE

**Fig. 2:**
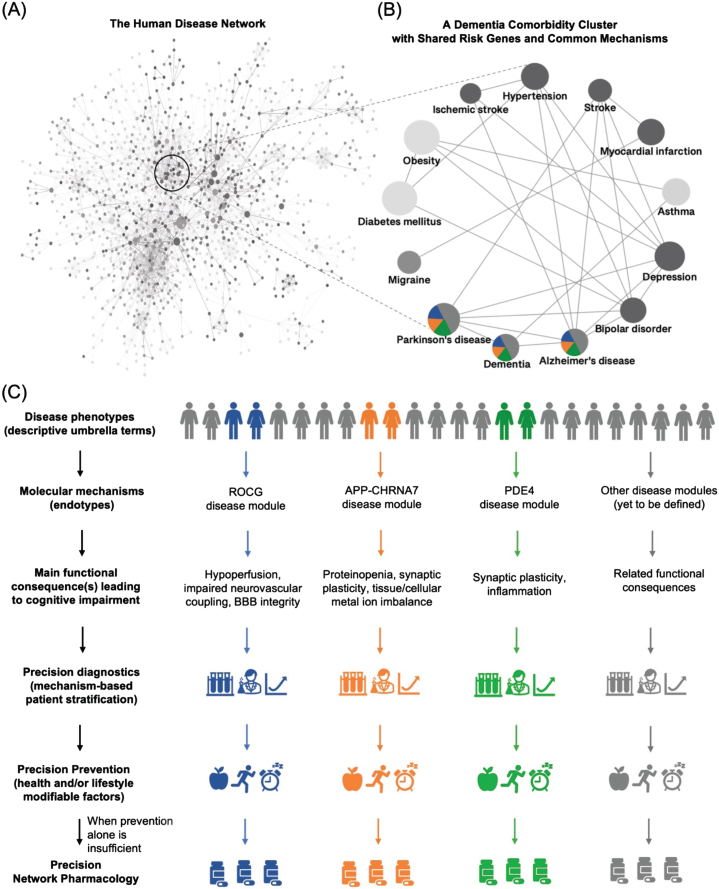
Schematic representation of a mechanism-based redefinition of dementia nosology for precision medicine. Dementia is an umbrella term, comorbid with several non-neurological disease phenotypes. Their associated shared risk genes point to mutual mechanistic endotypes. This then leads to molecular disease definitions allowing precision diagnosis and tailored therapeutic intervention. A) The Human Disease Network, the diseasome, adapted from [23] and first presented in 2007 by Goh et al. [17], was a landmark for the field of network medicine and disease research. It provided a novel perspective on the connections between various human diseases by their shared risk genes. B) A diseasome-derived cluster of heterogeneous phenotypes indicates shared mechanisms between dementias and other neurological and non-neurological disorders. C) A new framework to guide how we classify dementia by molecular endotypes, going from non-specific, homogenized umbrella terms to the identification of the underlying heterogeneous molecular mechanisms, followed by the development of mechanism-based diagnostic tools and precision and personalized treatment.

The network of all human diseases linked through shared risk genes, the diseasome, was a landmark demonstration of the similarities and overlaps between dementias and other neurological phenotypes to non-neurological disorders [29] (Fig. 2). Since then, the mechanistic relationships among this group of heterogenous phenotypes have been extensively validated by multiscale disease networks based also on shared genes, protein-protein interactions, drugs, symptoms, and comorbidities [30–32]. These mechanistic relationships thus provide the leads to identifying causative molecular mechanisms underlying different subtypes of dementia.

Systems and network medicine approaches enable the substitution of brain-centric umbrella definitions of disease, such as AD, by shared mechanistic endotypes. It is therefore useful to define endotype and phenotype beforehand for clarity. An endotype is a molecular mechanism underlying one or more phenotypes, which are, in turn, the functional (pathological) consequences, signs, or symptoms that can be observed in an individual. Current criteria for classifying dementias are phenotype-based, not endotype-based. This means that they are based on medical consensus about which signs and symptoms (such as brain atrophy, hyperintensities, tremor, memory loss, hallucinations, type of proteinopathy, etc.), age of onset, speed of progression, severity, or other descriptive features best describe a condition. However, different mechanistic endotypes can cause similar phenotypes of dementia but also different phenotypes in organs other than the brain. For example, AD is not only highly related at the causal level (shared risk genes) to other neurodegenerative diseases with common clinical features such as leukodystrophy and amyotrophic lateral sclerosis, but also to otherwise entirely distinct diseases such as polycystic ovary syndrome, which cannot be understood based on an organ-based disease taxonomy [33, 34]. These, and many other cases, are unlikely to represent “comorbidities” but different phenotypic expressions of the same common underlying mechanism, or endotype, in those affected. Exploiting this concept for precision medicine requires however, both the identification of potential endotypes and the availability of drugs that target the underlying causal mechanism in the relevant individuals, not organ-based symptom reduction across a clinically-diagnosed population.

It is worth acknowledging that the past decade has seen a marked increase in endotyping efforts aimed at addressing a wide range of complex diseases and syndromes in many other areas of medicine. These include, but are not limited to, cancer [35], coronary artery disease [36], asthma [37, 38], retinopathies [39, 40], chronic inflammatory diseases [41], sepsis [42], and sepsis in the context of COVID-19 [43].

## PRECISION ENDOTYPE-BASED DIAGNOSTICS

Endotype identification starts by the definition of disease modules. Currently, these can be best constructed from disease validated seed genes and their neighbors in the human protein-protein interaction network (*the interactome*). The resulting so-called protein-protein interaction modules, the disease modules, are the basis also for a mechanism-based redefinition of dementia and its endotypes. Though not all the interactions between human proteins are known and some of the known interactions are prone to false positives (i.e., experimental artifacts), the use of such disease modules on the interactome has provided valuable insights on the unifying biological processes and comorbidities across diseases and allowed the identification of candidate treatments targeting these modules [44, 45]. Recent advances in deep learning-based protein structure prediction [46] and large-scale sequencing and genome-wide association studies across individuals [47, 48] are expected to further improve the research gap in the characterization of protein interactions towards a more complete and refined disease module identification.

One example of such a disease module is related to reactive oxygen species (ROS) formation and cyclic guanosine monophosphate (cGMP) signaling (the ROCG disease module) [49]. A striking feature of this ROCG module is that it includes two of the most relevant AD risk genes, *PSEN2* and apolipoprotein E (*APOE*), and these interact directly with endothelial nitric oxide synthase (NOS3), a master modulator of vascular function. Indeed, non-physiological ROS formation and dysfunction of cGMP signaling have deleterious effects on the vasculature and are key mechanisms underlying pathophysiological features common to different types of dementia such as chronic hypoperfusion, impaired neurovascular coupling, and blood-brain barrier integrity. In addition, mechanistic preclinical studies show that disturbances of the functions of the proteins within the ROCG module are upstream events causally affecting traditionally dementia-associated markers such as tau [50, 51] and Aβ [52, 53]. Thus, when considering the hypothesis that not only this but many other disease modules may lead to tau and Aβ alterations widely found in the aged population, one can understand why clustering individuals based on such markers is preventing the identification of the true causes of dementia at the individual level.

## PRECISION NETWORK PHARMACOLOGY TARGETING MECHANISTIC ENDOTYPES IN DEMENTIA

Therapeutically, endotypes must not be reduced to the one-target-one-disease or “magic bullet” treatment approach. Disease modules are rather small localized multi-protein signaling networks, typically distinct from curated canonical signaling pathways. Currently known disease-gene associations that lay the foundation for describing disease modules typically reflect more common mechanisms across the general disease population. By incorporating the interactions of such common disease genes in the network together with omics data from patients, one can capture less frequently observed risk genes across individuals that are convergent at the pathway level and identify drug-target interactions for tailored therapy in patients [54–56].

In addition, a network needs network pharmacology, which is a different pharmacological approach from traditional polypharmacy and means targeting different proteins in the same disease module in a synergistic manner. Lack of a mechanism-based patient stratification and a single target approach, for example, may explain why the phosphodiesterase 5 (PDE5) inhibitor sildenafil, which potentially improves cGMP signaling within a defective ROCG module, did not decrease AD risk in general [57]. In addition, not all patients with AD dementia-like symptoms share the ROCG endotype. Therefore, this new mechanism-based diagnostic and therapeutic framework for dementia will be best tested in proof-of-concept adaptive clinical trials, where interventions align with the relevant mechanistic endotypes of those being treated [17].

In this context, numerous endotypes are yet to be defined and validated for future network pharmacology-based clinical trials. Potential new disease modules include other relevant dementia risk genes, and can be related for example to relevant loss of function of proteins such as AβPP, Aβ, other AβPP cleavage products (e.g., soluble AβPP alpha and AβPP-intracellular domain), and PSEN1 which have essential physiological roles in synaptic plasticity, hippocampal neurotransmitter release (e.g., soluble Aβ-mediated activation of α7-nicotinic acetylcholine receptors), as well as maintenance of metal ion homeostasis [58, 59]. An example of another potential endotype could be related to phosphodiesterase 4 (PDE4), with *PDE4B* [60] and *PDE4D* [61] gene variants reported to be associated with AD risk and disease progression. Interestingly, PDE4D is a direct neighbor of APP and bridging integrator 1 (BIN1), also an important AD risk gene, within the interactome, and its dysfunction is hypothesized to result in dysfunctional cAMP signaling affecting synaptic plasticity, cognition, regulation of inflammatory processes and tau pathology [62, 63]. The construction of these and further disease modules is the steppingstone towards a mechanism-based approach to dementia endotypes. Importantly, while in approximately 40% of the cases dementia can be potentially preventable by lifestyle interventions alone [64], network pharmacology stands to benefit from the increasing recognition of endotypes, disease modules, and their corresponding precision diagnostic tools to usher an era of precision medicine for patients with neurodegenerativedisorders.

## CONCLUSION

The path to precision clinical practice at the dawn of network medicine is challenging but achievable and starts with fundamentally rethinking how we define diseases. Throughout the last decade, network medicine has consistently demonstrated the pathobiological similarities among certain comorbidities (or phenotypes) and that these relationships can be exploited to identify disease modules (endotypes) and mechanism-based therapeutic strategies to treat these conditions despite current knowledge gaps or incomplete data [49]. The realization of such a conceptual revolution, of course, requires a matching level of methodological and technological readiness, including the assembly of phenotype-agnostic study cohorts, such as the CCBP, to determine endotypes of relevance to individuals with different phenotypes [65].

Above all, we need to recognize that we are dealing with a new discovery pipeline, one that necessarily must begin with genetics-based disease module discovery, not direct drug discovery, and the identification and validation of functionally meaningful and actionable (meaning treatable) molecular disease mechanisms, i.e., endotypes. Network-based approaches [66–69] offer the tools to systematically characterize the phenotypic heterogeneity observed in individuals with dementia [70, 71]. Accordingly, accounting for an individual’s comorbidities and specific patterns of neuroimaging, genetic, and molecular variability will be critical towards endotype-driven clinical decision making [72, 73]. Open challenges include the selection of only relevant and causal disease genes and reliable protein-protein interaction databases to construct the dementia-relevant signaling modules. Clinical diagnoses such as AD, PD, and other umbrella classifications, have clearly outlived their reductive usefulness for precision medicine. Their replacement with network medicine-derived endotypes, precision diagnosis and therapeutics will lead to the end of ‘dementia’ as we know it.
